# A comparison of pyogenic liver abscess in patients with or without diabetes: a retrospective study of 246 cases

**DOI:** 10.1186/s12876-018-0875-y

**Published:** 2018-10-01

**Authors:** Wenfei Li, Hongjie Chen, Shuai Wu, Jie Peng

**Affiliations:** Department of Infectious Diseases, Nanfang Hospital of Southern Medical University, Guangzhou, 510515 China

**Keywords:** Pyogenic liver abscess, Diabetes, Diagnosis, Treatment

## Abstract

**Background:**

Pyogenic liver abscess(PLA) has become common in patients with diabetes mellitus (DM), but it is unclear whether differences exist between patients with and without DM. A retrospective study was performed to identify these differences, summarize the clinical experience, and improve the diagnosis and treatment of PLA**.**

**Methods:**

The patients were enrolled in a teaching hospital from January 2012 to December 2016. The patients were separated into two groups based on comorbidity with diabetes mellitus (DM). The DM group was further separated into two subgroups according to the HbA1C concentration to investigate whether glycaemic control affected the clinical characteristics of PLA patients with DM. Chi-square, Fisher’s exact test, and t-tests were used to analyse and evaluate differences between the two groups.

**Results:**

Two hundred and forty-six PLA patients were identified and 90 (36.6%) had comorbid DM. Patients with DM were older, had higher levels of alkaline phosphatase and γ-glutamyl transferase, hypertension, a loss of body weight, a single abscess, and combined antibiotic therapy with the use of carbapenems and *Klebsiella pneumoniae* in their blood cultures but a less frequent history of abdominal surgery and *Escherichia coli* in their pus cultures. When DM patients were compared to non-DM patients, each of these differences was significant (*P* < 0.05). Diabetic PLA patients with poor glycaemic control had a significantly higher proportion of fever and both lobes abscess(*P* < 0.05).

**Conclusion:**

PLA patients with diabetes are older, have more serious complications, a higher prevalence of cardiovascular disease, an increased use of combined antibiotic therapy with carbapenem, and *K. pneumoniae* as the predominant pathogen, but these patients had fewer abdominal surgeries and fewer *E. coli* infections. In addition, poorly controlled glycaemia in diabetic PLA patients is associated with high incidence of fever and both lobes abscess.

## Background

Pyogenic liver abscess (PLA), which is a suppurating infection of the hepatic parenchyma, remains a condition associated with mortality and is reported in China and throughout the world, especially in Asia. The incidence rate of PLA is different worldwide and continues to increase annually [1]. In Taiwan, the annual all-age incidence of PLA has gradually increased from 10.83 to 15.45 cases per 100,000 individuals from 2000 to 2011 [2]. In northeast China, an incidence of 5.7 cases per 100,000 individuals was reported in a large population-based retrospective study [3]. In the United States, a large study described an incidence of 3.59 cases per 100,000 individuals [1]. PLA is concomitant with many diseases. These diseases are important risk factors and include diabetes mellitus (DM), malignancy, cholangitis, urinary tract disease, pneumonia, cardiovascular disease, autoimmune disease and malnutrition [1, 4–6]. In recent years, PLA patients with concomitant DM have become more common in this hospital, and previous case reports demonstrate that DM results in an increased risk of PLA [7]. Tian et al. provided a comprehensive perspective of PLA [8], but whether differences exist among PLA patients with and without concomitant DM is unknown, especially in South China. Furthermore, there is little information regarding the effects of glycaemic control on the characteristics of PLA in diabetic patients. In addition, it is unknown whether glycaemic control affects the clinical characteristics of PLA with DM. Therefore, the purpose of this investigation was to compare the clinical characteristics of PLA patients with and without concomitant DM, to investigate whether glycaemic control affects the clinical characteristics of PLA patients with DM and to improve the diagnosis and treatment of PLA.

## Methods

### Study population

All of the hospitalized patients diagnosed with PLA (International Classification of Disease, Clinical Modification 572.0) and treated at the Nanfang Hospital of Southern Medical University in Guangzhou, China, from January 2012 to December 2016 were enrolled. This hospital is a public-care, teaching-medical centre in Guangzhou that serves as a patient referral centre and accepts patient referrals from every part of Guangzhou. The diagnosis of PLA was based on the following criteria: 1) clinical features, such as fever, chills, abdominal fullness, and abdominal pain; 2)etiological tests of the blood and the abscess; and 3)imaging evidence of the abscess cavity in the liver as judged by abdominal ultrasonography (US), computerized tomography (CT), or magnetic resonance imaging (MRI). Patients were excluded who did not have clear records or did not complete the treatment. The included patients were divided into two groups: those with and those without DM. The criteria for type II DM were defined according to the 2017 standards [9]: 1)fasting plasma glucose (FPG) ≥ 126 mg/dL (7.0 mmol/L); 2)2-h plasma glucose (2-hPG) ≥ 200 mg/dL (11.1 mmol/L); 3) HbA1C ≥ 6.5% (48 mmol/mol); and 4) a random plasma glucose ≥200 mg/dL (11.1 mmol/L) in patients with classic symptoms of hyperglycaemia or hyperglycaemic crisis. To monitor glycaemic control, haemoglobin A1c (HbA1c) provides an estimate of average blood glucose during the preceding 3 months and is widely accepted as the primary indicator of the level of glycaemic control for the optimal management of diabetes [10]. The DM group was further categorised into two subgroups according to the HbA1C concentration: group 1, HbA1C < 7.0%, which indicated good glycaemic control, and group 2, HbA1C ≥ 7%, which indicated poor glycaemic control. The HbA1C cut-off value selection was based on previous studies [11].

### Data collection

Data were collected by reviewing the medical records of each patient. The records included demographic characteristics (age and sex), length of stay, hospital stay, duration aetiology, underlying diseases, clinical parameters (signs and symptoms), HbA1C levels, laboratory values (hematologic, biochemical, and microbiological findings), imaging features, diagnoses, antimicrobial therapy, catheter drainage, and outcomes at discharge (i.e., recovered or died).

### Statistical analysis

The statistical analysis was completed using the SPSS version 17.0 statistical software package(SPSS Inc., Chicago,Illinois, USA). All of the categorical variables were reported as percentages. The chi-square or Fisher’s exact test was applied to evaluate the differences in the categorical variables. Continuous data were presented as the mean with the standard deviation (SD) and the Student’s t-test was used to evaluate the differences in continuous variables. The statistical tests were performed with a two-tailed significance level of 0.05.

## Results

### Demographic characteristics

A total of 286 patients received hospital treatment for PLA during the study period. A total of 12 patients were excluded who did not fit the inclusion criteria, 18 were excluded whose medical treatment data were incomplete, and 10 were excluded who were transferred to another hospital before the completion of the treatment. Ultimately, 246 patients were included in this retrospective study. The demographic characteristics and clinical features of the PLA patients are shown in Table 1. We found that males were predominant (*n* = 160, 65%) and age ranged from 3 to 89 years with a mean age of 54.2 ± 14.2 years. The length of the hospital stay was 3 to 71 days with an average of 18.5 ± 11.4 days. Of the 246 patients, 90 (36.6%) had DM and 64 of these were men with a male-to-female ratio of 2.5:1.0. The mean age was 56.5 ± 10.9 years (26–84 years) for the DM group, which was higher than the 52.9 ± 15.6 years (3–89 years)for the non-DM group (*P* = 0.039). However, there was no difference in gender between the two groups (*P* = 0.129). Table 5 shows the clinical features in diabetic patients with good controlled or poorly controlled glycaemia. All of the diabetic patients had recorded HbA1C levels. Based on the HbA1C levels of the diabetic patients, 27 patients (30.0%) had good glycaemic control and 67 (70.0%) had poor glycaemic control. In our study, the age and gender were not identified between the controlled glycaemia groups (Table 5).

**Table 1:** Characteristics and clinical findings for patients with pyogenic liver abscess with diabetes mellitus (DM) or without DM (non-DM)

Characteristic	DM cases (%)	Non-DM cases (%)	*P* value (chi-square test)
Sex			
Male	64(71.1)	96(61.5)	0.129
Female	26(28.9)	60(38.5)	
Age (year) (mean ± SD)	56.5 ± 10.9	52.9 ± 15.6	0.039^a^
Underlying conditions			
Gallbladder diseases	42(53.3)	81(51.9)	0.427
Hypertension	24(26.7)	17(10.9)	0.001
Gastrointestinal surgery	3(3.3)	20(12.8)	0.025
Liver surgery	1(1.1)	14(9.0)	0.013
Pulmonary tuberculosis	3(3.3)	4(2.6)	1.000
Symptoms			
Fever	80(88.9)	138(88.5)	0.919
Chills	58(64.4)	93(59.6)	0.454
Abdominal pain	48(53.3)	90(57.7)	0.507
Frailty	33(36.7)	57(36.5)	0.984
Nausea or Vomiting	10(11.1)	23(14.7)	0.421
Cough	13(14.4)	17(10.9)	0.413
Weight loss	18(20.0)	13(8.3)	0.008

^a^Student’s t-test

### Underlying diseases

Although most patients in both groups had gallbladder diseases (*n* = 123, 50.0%) (i.e., gallstones, choledocholithiasis, chronic cholecystitis, pancreatitis, or postcholecystectomy), no significant differences were found between the groups. Hypertension (*n* = 41, 16.7%) was the second most common underlying disease and was more common in the DM group (26.7% vs. 10.9%, *P* = 0.001). This was followed by gastrointestinal surgery (*n* = 23, 9.3%), liver surgery (*n* = 15, 6.1%), pulmonary tuberculosis (*n* = 7, 2.5%), nephrotic syndrome (n = 1, 0.4%) and hyperthyroidism (n = 1, 0.4%). Patients with DM had a lower prevalence of gastrointestinal surgery (3.3% vs. 12.8%, *P* = 0.025) and liver surgery (1.1% vs. 9.0%, *P* = 0.027) (Table 1). However, the underlying disease did not differ between the good controlled and poorly controlled glycaemia groups (Table 5).

### Clinical features

In both groups, the most common symptom was fever (*n* = 218, 88.6%) with 88.9% in the DM group and 88.5% in the non-DM group; this symptom was followed by chills (*n* = 151, 61.4%), abdominal pain (*n* = 138, 56.1%), frailty (*n* = 90, 36.6%), nausea or vomiting (*n* = 33, 13.4%), weight loss (*n* = 31, 12.6%), cough (*n* = 30, 12.2%), abdominal fullness (*n* = 8, 3.3%) and jaundice (*n* = 5, 2.0%) in decreasing order. The DM group had a higher prevalence of body weight loss (20.0% vs. 8.3%, *P* = 0.008) (Table 1), but there were no significant differences among the glycaemic control groups. However, patients with poorly controlled glycaemia had a higher rate of fever (77.8% vs. 93.7%, *P* = 0.028) (Table 5).

### Laboratory examination

In both groups, inflammatory biomarkers were generally elevated and included C-reactive protein (CRP), procalcitonin (PCT), erythrocyte sedimentation rate (ESR), and leukocyte and neutrophil count. Remarkably, the sensitivity of the erythrocyte sedimentation rate was 100% positive in all of the PLA patients. In addition, C-reactive protein and procalcitonin were approximately 100% positive in all PLA patients. A higher incidence of elevated alanine aminotransferase (ALT), aspartate aminotransferase (AST), total bilirubin (T.Bil), alkaline phosphatase (ALP), γ-glutamyl transferase (GGT) and decreased albumin (ALB)levels were detected in both groups. Additionally, patients with DM had a higher prevalence of ALP (73.5% vs. 49.2%, *P* = 0.020) and GGT(91.2% vs. 46.2%, *P* = 0.001) (Table 2). However, no laboratory examination was identified in the group with controlled glycaemia (Table 5).

**Table 2:** Laboratory and image findings for pyogenic liver abscess patients with diabetes mellitus (DM) or without DM (non-DM)

Characteristic	DM cases (%)	Non-DM cases (%)	*P* value (chi-square test)
Laboratory findings			
WBC > 9.5 (× 10^9/L)	64/90(71.1)	96/156(61.5)	0.129
WBC < 3.5 (× 10^9/L)	1/90(1.1)	4/156(2.6)	0.397^a^
NEUT> 75%	62/90(68.9)	101/156(64.7)	0.508
Anaemia^b^	71/90(78.9)	113/156(72.4)	0.262
PLT > 350(× 10^9/L)	28/90(31.1)	59/156(37.8)	0.289
PLT < 125(× 10^9/L)	12/90(13.3)	23/156(14.7)	0.760
↑ESR(mm/1 h)	27/27(100)	32/32(100)	1.000
CRP > 5(mg/L)	81/83(97.6)	131/137(95.6)	0.449
PCT > 0.05(ng/ml)	66/66(100)	90/92(97.8)	0.510^a^
T.Bil > 20.5(μmol/L)	29/89(32.6)	39/155(25.2)	0.468
ALT> 50(U/L)	32/90(35.6)	54/155(34.8)	0.910
AST > 40(U/L)	27/90(30.0)	57/154(37.0)	0.266
ALP > 125(μmol/L)	25/34(73.5)	32/65(49.2)	0.020
GGT > 60(U/L)	31/34(91.2)	30/65(46.2)	0.001
ALB< 40(g/L)	87/90(96.7)	142/155(91.6)	0.123
Abscess location			
Right lobe	63/90(70.0)	108/156(69.2)	0.232
Left lobe	16/90(17.8)	29/156(18.6)	
Both lobes	9/90(10.0)	19/156(12.2)	
Caudate lobe	2/90(2.2)	0(0)	
Abscess size (cm)			
< 5	21/90(23.3)	37/156(23.7)	0.907
5–10	49/90(54.5)	88/156(56.4)	
> 10	20/90(22.2)	31/156(19.9)	
Count of abscess			
Single	75/90(83.3)	110/156(70.5)	0.025
Multiple	15/90(16.7)	46/156(29.5)	

*WBC* white blood cell count, *NEUT* neutrophil count, *PLT* platelets, *ESR* erythrocyte sedimentation rate, *PCT* procalcitonin, *CRP* C-reactive protein, *ALT* alanine aminotransferase, *AST* aspartate aminotransferase, *T.Bil* total bilirubin, *ALP* alkaline phosphatase, *GGT* γ-glutamyl transferase, *ALB* albumin

^a^Fisher’s exact test; ^b^ Haemoglobin < 130 g/l in men, < 115 g/l in women; ↑ESR > 15 mm/1 h in men, > 20 mm/1 h in women

### Imaging

All of the cases were imaged by ultrasonography, computerized tomography (CT), or magnetic resonance imaging (MRI), but no significant difference was found between the groups. According to these images, most of the lesions (*n* = 171, 69.5%) were located in the right lobe (70.0% in the DM group and 69.2% in the non-DM group) and the diameters ranged from 0.8 × 0.6 cm to 19.0 × 17.7 cm. Patients with a single abscess (*n* = 185, 75.2%) were three-fold as common as patients with multiple abscesses (*n* = 61, 24.8%), and the DM group had a higher prevalence of a single abscess(83.3% vs. 70.5%, *P* = 0.025); however, the difference was not statistically significant for either abscess lesion number or size (Table 2). Remarkably, patients with poor glycaemic control had a higher prevalence in both lobes and the left lobe but less in the right lobe abscess(14.3% vs. 0, 23.8% vs. 3.7%, and 60.3% vs. 92.6%, respectively, *P* = 0.010) (Table 5).

### Aetiology

Blood cultures were collected from 118 patients and 24.6% (29 cases) were positive. The DM group had a higher positive proportion(36% vs. 16.2%, *P* = 0.013). Only 6.9%(two cases)of the culture-positive patients had polymicrobial growth. Pus cultures were collected from 121 patients and the overall positive growth rate was 58.7% (71 cases). Only 4.2%(three cases)had polymicrobial growth. A total of 9 cases were positive for both blood and pus cultures. A total of 5 cases had monomicrobial infections and 4 cases had polymicrobial infections. Among the culture-positive patients, 105 strains were identified, which included 10 Gram-positive organisms (9.5%) and 95 Gram-negative organisms (90.5%). *Klebsiella pneumoniae* was the most common pathogen identified in both blood and pus cultures for both groups and had a higher prevalence in blood cultures(26% vs. 1.5%, *P* = 0.001). The second most common pathogen was *Escherichia coli*, which was less frequently isolated from pus cultures of the DM group(16% vs. 2.2%, *P* = 0.037). Other pathogens were isolated from < 5% of the patients (Table 3). However, *K. pneumoniae* infections and *E. coli* infections showed no significant differences between the glycaemic control groups in our study (Table 5).

**Table 3:** Microbiological isolates in blood and pus cultures from patients with diabetes mellitus (DM) or without DM (non-DM)

Characteristic	Blood culture			Pus culture			Strains
	DM (*n* = 50)	Non-DM (*n* = 68)	*P* value (chi-square)	DM (*n* = 46)	Non-DM (*n* = 75)	*P* value (chi-square)	
Positive growth	18	11	0.013	28	43	0.701	–
Polymicrobial growth	1	1	1.000	1	2	1.000	–
Gram-positive aerobes							10 (9.5)
*Staphylococcus*	2	0	0.177^a^	1	2	1.000	5(4.7)
*Streptococcus*	1	0	0.424^a^	0	2	0.525^a^	3 (2.9)
*Enterococcus*	0	2	0.507^a^	0	0	–	2 (1.9)
Gram-negative organisms							95 (90.5)
*Klebsiella pneumonia*	13	1	0.001	23	25	0.069	62 (59.0)
*Escherichia coli*	2	7	0.357	1	12	0.037	22 (21.0)
*Pseudomonas*	0	1	1.000^a^	0	1	1.000^a^	2 (1.9)
*Aeroenterobacter*	0	0	–	2	1	0.665	3(2.9)
*Burkholderia cepacia*	1	0	0.424^a^	1	0	0.380^a^	2 (1.9)
*Acinetobacter baumannii*	0	0	–	1	0	0.380^a^	1(0.95)
*Enterobacter cloacae*	0	0	–	0	1	1.000^a^	1(0.95)
*Bacillus citrate*	0	1	1.000^a^	0	0	–	1(0.95)
*Shewanella putrefaciens*	0	0	–	0	1	1.000^a^	1(0.95)

^a^Fisher’s exact test

### Treatments and outcomes

Antibiotic therapy was the most common treatment for both groups (*n* = 241, 98.0%), which included 46 patients (19.1%) who received a single antibiotic and 195 patients (80.9%) who received a combination antibiotic therapy. The remaining five patients only accepted simple abscess drainage. In both groups, the most frequently used antibiotics were third generation cephalosporins (including ceftriaxone, ceftazidime, or cefoperazone) (*n* = 167, 69.3%), which were followed by fluoroquinolone (including levofloxacin, moxifloxacin, or ciprofloxacin) (*n* = 72, 29.9%), carbapenems (imipenem or meropenem)(*n* = 64, 26.6%), or combined with metronidazole (*n* = 109, 45.2%). It is notable that compared to the non-DM group, the DM group had a significantly higher frequency of combined antibiotic therapy (86.7% vs. 75.0%, *P* = 0.009) with carbapenems (36.7% vs. 19.9%, *P* = 0.004). In addition, percutaneous drainage was performed in 134 (54.5%) patients and surgical drainage was performed in 22 patients (8.9%), but there were no differences between the groups. The total effective rate of the therapy was 96.3%(237/246) and the two groups had similar rates. A total of 6 cases were invalid and 3 cases died from septic shock or multiple organ failure (MOF). No significant difference was noted in the ratio of the effective treatment and the mortality between the groups (Table 4). Furthermore, the treatment strategy, hospitalization days and mortality were not significantly different between the controlled glycaemia groups either (Table 5).

**Table 4:** Treatment and outcome in pyogenic liver abscess patients with diabetes mellitus (DM) or without DM (non-DM)

Characteristic	DM cases (%)	Non-DM cases (%)	*P* value (chi-square test)
Antibiotic option			
Combined	78(86.7)	117(75.0)	0.009
Single	9(0.1)	37(23.7)	
Antibiotic drugs			
The third generation of cephalosporin	63(70.0)	104(66.7)	0.590
Fluoroquinolone	33(36.7)	39(25.0)	0.053
Carbapenems	33(36.7)	31(19.9)	0.004
Metronidazole	43(47.8)	66(42.3)	0.405
Method of abscess drainage			
Percutaneous drainage	51(56.7)	83(53.2)	0.599
Surgical drainage	7(7.8)	15(9.6)	0.627
Clinical outcomes			
Cured	85(94.4)	152(97.4)	0.395
Death	1(1.1)	2(1.3)	1

**Table 5:** Baseline characteristics, clinical presentation, and outcome of diabetic patients with good or poorly controlled glycaemia

Characteristic	Good control of glycaemia (*n* = 27)(%)	Poor control of glycaemia (*n* = 63)(%)	*P* value (chi-square test)
Male	20 (74.1)	44 (69.4)	0.685
Age (year) (mean ± SD)	57.2 ± 2.0	56.1 ± 1.4	0.681
Underlying conditions			
Gallbladder diseases	15 (55.6)	27 (42.9)	0.268
Hypertension	7 (25.9)	17 (26.9)	0.917
Abdominal surgery	2 (7.4)	1 (1.6)	0.159
Liver surgery	0 (0)	1 (1.6)	0.510
Pulmonary tuberculosis	1 (3.7)	2 (3.2)	0.909
Symptoms			
Fever	21 (77.8)	59 (93.7)	0.028
Chills	14 (51.9)	44 (69.8)	0.102
Abdominal pain	14 (51.9)	34 (54.0)	0.854
Weight loss	5 (18.5)	13 (20.6)	0.818
Laboratory findings			
WBC > 9.5 (×10^9/L)	18/27 (66.7)	46/63 (73.0)	0.543
NEUT> 75%	16/27 (59.3)	46/63 (73.0)	0.196
Anaemia^b^	22/27 (81.5)	49/63 (77.8)	0.693
PLT > 350(×10^9/L)	3/27 (11.1)	9/63 (14.3)	0.946
PLT < 125(×10^9/L)	11/27 (40.7)	17/63 (27.0)	0.196
↑ESR(mm/1 h)	7/7 (100)	20/20 (100)	1.000
CRP > 5(mg/L)	25/25 (100)	56/58 (96.6)	1.000^a^
PCT > 0.05(ng/ml)	20/20 (100)	46/46 (100)	1.000
T.Bil > 20.5(μmol/L)	11/27 (40.7)	18/62 (29.0)	0.279
ALT> 50(U/L)	11/27 (40.7)	21/63 (33.3)	0.501
AST > 40(U/L)	9/27 (33.3)	18/63 (28.6)	0.919
ALP > 125(μmol/L)	6/10 (60)	19/24 (79.2)	0.467
GGT > 60(U/L)	9/10 (90)	22/24 (91.7)	1.000^a^
ALB< 40(g/L)	26/27 (96.3)	61/63 (96.8)	1.000^a^
Abscess locations			
Right lobe	25 (92.6%)	38 (60.3%)	0.010
Left lobe	1 (3.7%)	15 (23.8%)	
Both lobes	0 (0)	9 (14.3%)	
Caudate lobe	1 (3.7%)	1 (1.6%)	
Abscess size (cm)			
< 5	4 (14.8)	16 (25.4)	0.526
5–10	16 (59.3)	34 (54.0)	
> 10	7 (25.9)	13 (20.6)	
Multiple abscesses	4 (14.8)	11 (17.5)	0.758
*K. Pneumonia* infections	6/18 (33.3)	25/50 (50)	0.223
*E.coli* infections	1/18 (5.6)	2/50 (4)	1.000^a^
Treatment			
Percutaneous drainage	15 (55.6)	36 (57.1)	0.889
Surgical drainage	2 (7.4)	5 (7.9)	0.932
Antibiotics only	10 (37.0)	22 (35.0)	0.848
Clinical outcomes			
Cured	27 (100)	58 (92.1)	0.132
Death	0 (0)	1 (1.6)	1.000^a^
Hospitalization days	22.3 ± 2.3	19.4 ± 1.3	0.252

*WBC* white blood cell count, *NEUT* neutrophil count, *PLT* platelets, *ESR* erythrocyte sedimentation rate, *PCT* procalcitonin, *CRP* C-reactive protein, *ALT* alanine aminotransferase, *AST* aspartate aminotransferase, *T.Bil* total bilirubin, *ALP* alkaline phosphatase, *GGT* γ-glutamyl transferase, *ALB* albumin

^a^Fisher’s exact test; ^b^ Haemoglobin < 130 g/l in men, < 115 g/l in women; ↑ESR > 15 mm/1 h in men, > 20 mm/1 h in women

## Discussion

The morbidity of patients with PLA and diabetes has recently increased. This may be due to reduced immunity, neutrophil chemotaxis, mononuclear phagocyte activation, and/or opsonization in diabetes patients. In addition, hyperglycaemia can promote bacterial growth in tissues, and metabolic disorders impact the liver, gut, pancreas, stomach, and intestine, which induces biliary disease. DM is a risk factor for PLA with a hazard risk rate of 3.6 to 9-fold [6, 7] and it is relatively common in PLA patients with reported co-existence rates of 30% in Hong Kong [12], 31% in Canada [13], 28.7% in a single centre in Xi’an, China [14], 23% in Italy [15], and 36.6% in this study.

The majority of the patients in this study were males with a mean of 54.2 ± 14.2 years old, which is comparable with other studies [16]. Patients with DM were approximately 4 years older than patients without DM, which was similar to previous reports [17] and may relate to the fact that most DM patients are older and immunocompromised. Notably, biliary tract diseases were the major underlying disease process, which indicated that biliary infections were the predominate cause of PLA, and this is consistent with a previous study from east China [18]. In addition, patients with DM had a higher prevalence of hypertension, which suggests that PLA patients with DM were more likely to have cardiovascular disease. In this investigation, gastrointestinal operations included appendectomy (*n* = 10), enterectomy (*n* = 7), laparotomy (*n* = 4) and hemigastrectomy (*n* = 2), and were more common in the non-DM group, which indicated that the gastrointestinal operation history for PLA patients without diabetes was relevant, especially for appendectomy [19]. In our study, liver surgery included a partial hepatectomy (*n* = 11), transcatheter arterial chemoembolization (TACE) (*n* = 3) and splenectomy (n = 1). Although no significant difference was found between the groups, TACE and splenectomy have been linked with certain PLA, which were statistically significant independent risk factors [20, 21]. In addition, patients without DM have been reported to have a higher prevalence of biliary tract diseases [17], but this investigation failed to support that finding.

In our study, the main clinical findings for PLA patients were fever, chills, and abdominal pain, which is consistent with other studies [13, 16–18, 22]. Clinical features, such as cough, jaundice, frailty, and abdominal fullness are not typical and may relate to a delayed diagnosis of PLA. The loss of body weight was higher in the DM group and this may be because DM patients ineffectively use glucose, which results in increased consumption of body fat and protein, and this metabolic condition may enhance more infection. In addition, we found that poorly controlled glycaemia patients were prone to have a fever, which is different from previous studies [23] indicating that glycaemia control affects the severity of PLA.

The laboratory outcomes did not differ between the groups. Most patients had elevated white blood cell counts (WBC), neutrophil counts, C-reactive protein (CRP), procalcitonin (PCT), erythrocyte sedimentation rate (ESR) levels, and abnormal liver function tests, but there was no significant difference between the groups. The CRP, PCT and ESR levels appeared to be more sensitive than WBC. Collectively, the analysis of these biomarkers may reduce the misdiagnosis of PLA. Furthermore, the alkaline phosphatase and γ-glutamyl transferase levels of the DM group were higher, which suggests that liver injury in PLA patients with DM was more remarkable with more fatty liver and biliary cell damage. Nevertheless, our study showed that no laboratory examination was significantly identified between good and poorly controlled glycaemia.

We all know that imaging is crucial for the diagnosis of PLA. In this investigation, most liver abscess was singular and located in the right lobe with a diameter of 5–10 cm, which was in agreement with previous studies [14, 17]. This may be due to the large area of the right liver lobe and its propensity to receive the most portal blood flow [17]. Interestingly, unlike the non-DM group, the overwhelming majority of DM patients had a single abscess. This may be because patients with non-DM had a higher incidence of previous surgeries with possible abdominal infection involving other areas of the liver. It is also interesting to note that patients with poorly controlled glycaemia had a higher rate of both lobes. This may be because poorly controlled glycaemia can help bacteria grow, which makes the overall condition worse. Thus, it is important to control good glycaemia.

Gram-negative bacteria predominated in this investigation, which indicates that antibiotics against Gram-negative bacteria should be used for empiric therapy. In addition, we also found that *K. pneumoniae* was the dominant pathogen (accounting for 59.0% of the pathogens), which is consistent with previous studies from Asian countries where it accounted for 40–80% [16, 24–26]. Followed by *E. coli,Staphylococcus*, *E. aerogenes*, and *Streptococcu*s. In this investigation, the prevalence of *K. pneumoniae* in blood cultures was higher in the DM group. These findings are consistent with previous studies and could be explained by possible intimal vascular defects in DM patients [17]. In contrast, PLA patients without DM had a higher prevalence of *E. coli* in pus cultures, which is similar to a previous study by Tian et al. [8], which may be correlated with a higher incidence of post-abdominal operation and resulting liver infection. Remarkably, the incidence of positive pus cultures was similar between the two groups. Positive pus cultures were significantly higher than blood cultures, which is similar to a previous report [8, 14]. These findings imply that bacteria were primarily confined to the liver. The administration of antibiotics and anti-fever drugs was significantly lower than the incidence of positive blood cultures. Positive blood cultures were higher in the DM group, which suggests that PLA patients with DM are more likely to develop blood infections or even sepsis. Previous studies have shown that diabetic PLA patients with poor glycaemic control had higher *K. pneumoniae* infection rates [23], but our study failed to support this finding. In our study, 98.0% of the patients underwent antibiotic therapy, 54.5% underwent percutaneous drainage, and 8.9% underwent surgical drainage, which resulted in an effective rate of 96.7% and a fatality rate of 1.2%, which was similar to recent international reports of 0.9–2.5% [5, 16, 18]. For treatment, third generation cephalosporin or fluoroquinolone combined with metronidazole was used. Carbapenems were used if the outcomes were not optimal or if patients were in a critical condition. Remarkably, 5 patients presented with severe sepsis and 4 patients required an ICU stay and they all received combination antibiotic therapy including carbapenems for broad coverage. It is notable that the DM group had a higher proportion of antibiotic combined therapy and carbapenems and a higher likelihood of severe complications with difficult to control infections. These observations suggest that PLA patients with DM may need more aggressive combined therapy with carbapenems. However, there was not a significant difference between percutaneous drainage and surgical therapy for the two groups. Our study showed that the treatment strategy, hospitalization days and mortality were not significantly different between the controlled glycaemia groups. Based on this investigation, antibiotic therapy and catheter drainage are the appropriate treatments for PLA patients with or without DM.

There were notable limitations to this study. This was a retrospective study, it was performed in a single centre, and the results may not be generalizable. However, the results are based on a large number of cases and should be valuable to other investigators and clinicians.

## Conclusions

In conclusion, PLA was mainly due to biliary tract disease with a single lesion located in the right lobe, and the predominant pathogen was *K. pneumonia*. PLA patients with and without DM had many differing clinical characteristics. PLA patients with DM were older and had more complications including a higher prevalence of cardiovascular disease, a loss of body weight, *K. pneumonia* infections, antibiotic combined therapy with carbapenem, and a greater likelihood of sepsis. In contrast, a history of gastrointestinal surgery and *E. coli* were less frequent. Furthermore, diabetic PLA patients with poor glycaemic control had a significantly higher proportion of fever and both lobes abscess. Additional large-scale studies and fundamental research can build upon this investigation and should provide further insight into PLA.

## Abbreviations

2-hPG: 2-h plasma glucose
ALB: Albumin
ALP: Alkaline phosphatase
ALT: Alanine aminotransferase
AST: Aspartate aminotransferase
CRP: C-Reactive protein
CT: computerized tomography
DM: Diabetes mellitus
*E. coli*: *Escherichia coli*
ESR: Erythrocyte sedimentation rate
FPG: Fasting plasma glucose
GGT: γ-Glutamyl transferase
MRI: Magnetic resonance imaging
NEUT: Neutrophil count
PCT: Procalcitonin
PLA: Pyogenic liver abscess
PLT: Platelets
SD: Standard deviation
T.Bil: Total bilirubin
US: Ultrasonography
WBC: White blood cell count
